# Intelligent lecture recording system based on coordination of face-detection and pedestrian dead reckoning

**DOI:** 10.7717/peerj-cs.971

**Published:** 2022-05-17

**Authors:** Hu Wang, Jianpeng Hu

**Affiliations:** School of Electronic and Electrical Engineering, Shanghai University of Engineering Science, Shanghai, Shanghai, China

**Keywords:** Face detection, Particle filter, Wireless communication, Pedestrian dead reckoning (PDR)

## Abstract

Automatic lecture recording is an appealing alternative approach to manually recording lectures in the process of online course making as it can to a large extent save labor cost. The key of the automatic recording system is lecturer tracking, and the existing automatic tracking methods tend to lose the target in the case of lecturer’s rapid movement. This article proposes a lecturer tracking system based on MobileNet-SSD face detection and Pedestrian Dead Reckoning (PDR) technology to solve this problem. First, the particle filter algorithm is used to fuse the PDR information with the rotation angle information of the Pan-Tilt camera, which can improve the accuracy of detection under the tracking process. In addition, to improve face detection performance on the edge side, we utilize the OpenVINO toolkit to optimize the inference speed of the Convolutional Neural Networks (CNNs) before deploying the model. Further, when the lecturer is beyond the camera’s field of view, the PDR auxiliary module is enabled to capture the object automatically. We built the entire lecture recording system from scratch and performed the experiments in the real lectures. The experimental results show that our system outperforms the systems without a PDR module in terms of the accuracy and robustness.

## Introduction

Various online courses have emerged as information technology in education develops rapidly. Significantly, the advantages of the online teaching modes are gradually showing under the case of COVID-19 outbreak in recent years. Although the rapid development of computer technology has made it possible to provide richer educational resources online, the cost of designing and developing multimedia teaching facilities is still very high (Zhang et al., 2021). Lecture recording plays an important role in online learning and distance education. Most of the videos on online course platforms are recorded by technical experts in professional studios using expensive photographic equipment (Tan, Kuo & Liu, 2019). Moreover, the core of the online video recording is the camera operated by professional videographers (Lalonde et al., 2010), which causes the large consumption of human-power. Hence, automatically detecting the lecturer and recording video is necessary from a cost point of view.

To produce high-quality lecture recordings using an automatic recording system, artificial intelligence technology is needed to direct the camera’s rotation because speakers move around a great deal during their presentations. Using a fixed camera limits the lecturer’s range of movement and requires human intervention during the recording process. Using a Pan-Tilt (PT) camera is more conducive to expanding the capture range. Some automatic recording systems are designed to capture the lecturer *via* an automatic PT camera (Zawadzki & Gorgoń, 2015). However, in reality, the sudden movements of the lecturer often lead to tracking failures with such cameras. To solve these problems, we investigated the use of a PT camera enhanced with extra localization technology for the real-time tracking of the lecturer, even if it moved quickly beyond the camera’s field of view.

This PT camera-based solution integrates two types of core technology. First, a deep neural network is used to track the lecturer, and the camera is panned to capture the lecturer. Second, sensor-based localization technology is used to identify the lecturer’s position.

In the field of object detection and tracking, Convolutional Neural Networks (CNNs) have been widely used in computer vision applications (Wu et al., 2017), and its effect is greatly superior to that of traditional detection methods, such as Fast R-CNN (Girshick, 2015). CNNs have proved to be superior in terms of speed and accuracy when tested in challenging benchmarks. However, their application in realistic scenarios has not been ideal. For example, SiamFC (Bertinetto et al., 2016) and a series of Siamese networks (Zheng et al., 2020) can automatically find and mark a target with a bounding box after detecting it in the first frame; however, this method usually depends on the computer’s performance because of its enormous complexity. Mobilenets (Howard et al., 2017), as a representative of lightweight networks, provides a lightweight model by replacing the traditional convolutional model with depth-separable convolution. It can reduce the number of operations and weight parameters of the network. Here we introduce a lightweight detection network that ensures target detection speed and reduces dependence on computer performance. It can satisfy the requirements of a real-time recording system deployed on normal-performance computers. Existing localization technologies usually require special photographic equipment, wireless signal devices, and extra sensors. Therefore, setting up an intelligent classroom, for example, with a panoramic camera (Sun et al., 2020) and a multi-camera system (Taj & Cavallaro, 2011) can be costly.

Even if all this technology is in place, the results are often suboptimal when using wireless devices with limited system resources, the collection of offline information is subpar, and indoor Wi-Fi positioning is used (Boonsriwai & Apavatjrut, 2013). In 2019, Tan, Kuo & Liu (2019) proposed a method that combined face detection with infrared (IR) thermal sensors, which are sensitive to temperature. However, for sensor-based indoor localization methods, the critical task is to estimate the target’s step length and angle (Harle, 2013). Smartphones are the most widely used portable devices that contain an inertial measurement unit (IMU). Thus, there is much interest in developing new indoor positioning solutions based on IMUs within smartphones.

The most common indoor positioning method is pedestrian dead reckoning (PDR). Although, PDR suffers from problems, such as the generation of cumulative errors and inaccurate step length estimation (Li et al., 2021). Therefore, we have introduced a particle filtering algorithm to improve PDR positioning accuracy. Particle filtering has been widely used in statistics (Xiong et al., 2021), computer vision (Kong et al., 2021), signal processing (Saha, Bambha & Bhattacharyya, 2010), and other fields. It has also been further extended to solve nonlinear, non-Gaussian Bayesian non-recursive filtering problems. Wu et al. (2016) used particle filtering technology to fuse Wi-Fi fingerprint and inertial sensor information and perform optimization processing to achieve high-precision positioning.

This article proposes a portable, accurate, and low-cost intelligent lecture recording system that automatically tracks lecturers. Smartphone sensors are used to detect the lecturer’s position and retrieve lost targets, and the system employs an edge-side deployable lightweight network. The PDR module is based on particle filtering and effectively detects the lecturer’s position. The accurate face detection model ensures that the lecturer’s face is in the center of the screen when they appear on the screen.

Hence, the three major contributions of this work are as follows:

1. The system can automatically track and update the lecturer’s location information in real time. It is also very robust and easy to deploy.
2. The PDR module can obtain acceleration information from the smartphone sensor to predict the lecturer’s position. To prevent detection failure caused by rapid movements and background interference in face detection, the PDR module can help the system locate the lecturer when the camera loses its target.
3. The particle filtering algorithm fuses the landmark point messages and PDR information, and the landmark point corresponding to the PT camera’s angle of rotation is used as the observation data. Consequently, localization accuracy is improved, which solves the problem of quick error accumulation for the PDR module.

## Related work

In the field of lecturer tracking systems, the detection of objects in video sequences has been a hot research topic in recent years. Traditionally, only image information has been considered, which can result in the lecturer moving out of the camera’s field of view and the system not being able to track them.

Winslow et al. (2009) improved access to recorded lecture content from mobile devices. They established a method that enabled the incorporation of exterior slides into lecture recordings, which made it more feasible to record educational videos. In addition, devices that provide depth information, such as Kinect (Kamezaki et al., 2014), can be utilized to retrieve more reliable target scenes to improve the tracking performance of Pan-Tilt-Zoom (PTZ) cameras. JiuHong et al. (2009) proposed a system that locates the lecturer *via* a wireless portable microphone. This led to the generation of more comprehensive recordings with rich educational content: the lecturer’s speech, the lecture notes, the computer courseware, the lecturer’s interactions with students, and the students’ actions. However, a multi-camera system is required to capture the different types of content. Liao et al. (2015) then designed a lecture recording system that uses a single PTZ camera. The system simultaneously focuses on the lecturer and handwritten content, and the camera focuses mainly on the handwriting area.

Wang & Mei (2013) developed a monocular active vision module to track lecturers in real time. They utilized a face recognition method based on AdaBoost and a random learning tracking method to generate a module that can robustly track the lecturer’s face. However, AdaBoost is insensitive to changes in scale. Shavalipour & Haris (2014) used detection-based algorithms to estimate object location in every frame independently. This involved applying multiple detectors to locate multiple body parts to reduce tracking failures. However, this approach requires offline data for training and cannot identify untrained samples. Tan, Kuo & Liu (2019) proposed a face detection method that uses IR thermal sensors. An advantage of this method is that IR sensors can determine the lecturer’s position when the lecturer moves abruptly. A disadvantage is that IR sensors are easily affected by ambient temperature.

Recently, there has also been considerable interest in developing a smartphone-based system that can locate a person in an indoor environment (Seco & Jiménez, 2018). Yang et al. (2016) proposed a fuzzy inference system that achieves highly accurate tracking by using inertial sensors. Nevertheless, it can only run in the short-term.

### Proposed auxiliary positioning method

**Figure 1:**
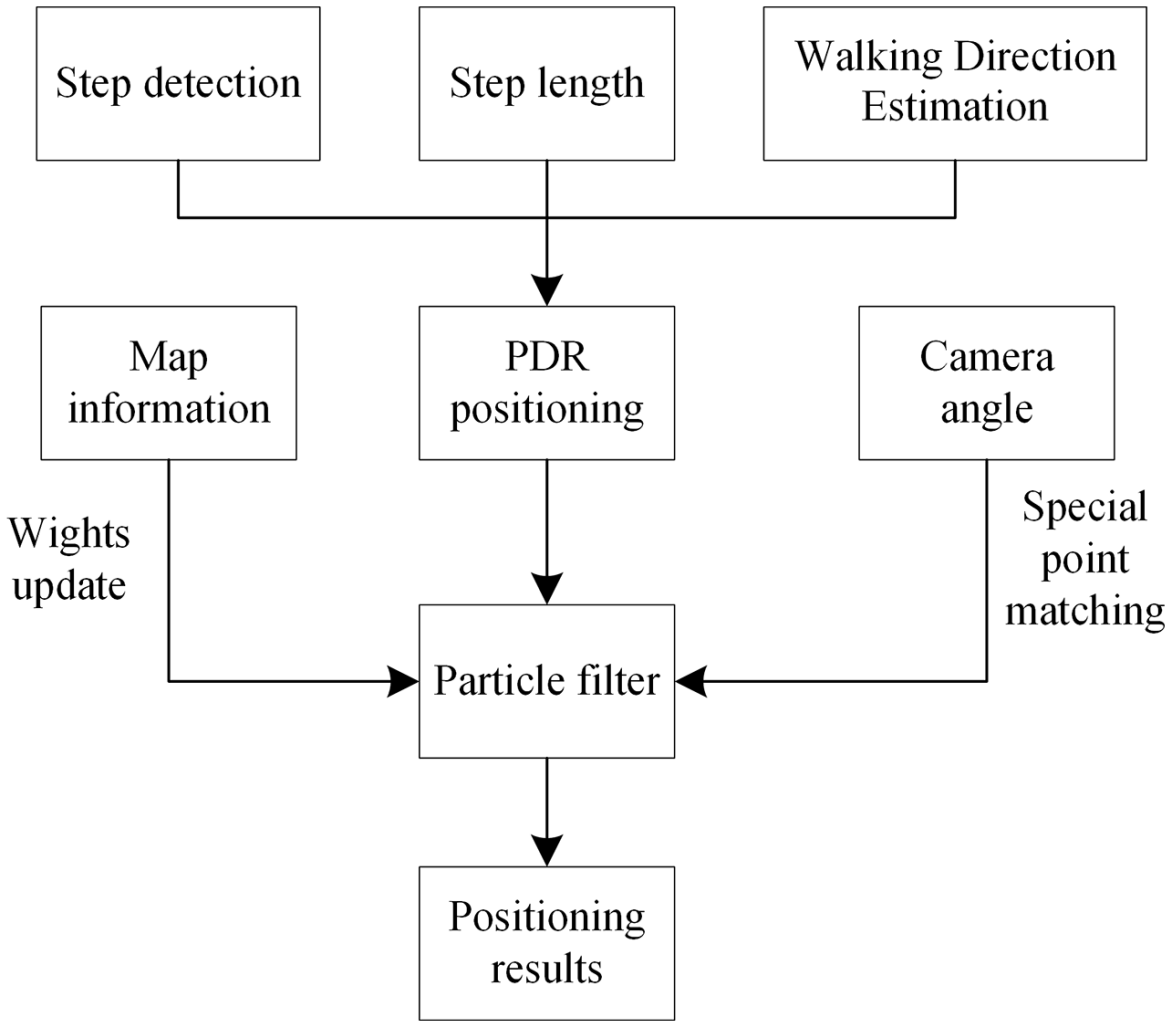
Positioning method based on particle filter.

In this section, the auxiliary positioning method based on PDR and particle filtering is explained. A particle filter algorithm has been exploited to fuse rotation information with PDR information. The lecturer’s position is estimated using the PDR step length and heading angle information, and particle filtering is used to fuse the map and camera angle information. This positioning method, based on information fusion and particle filtering, is shown in Fig. 1. This study has been approved by the school of electronic and electrical engineering, Shanghai university of engineering science.

### PDR positioning

**Figure 2:**
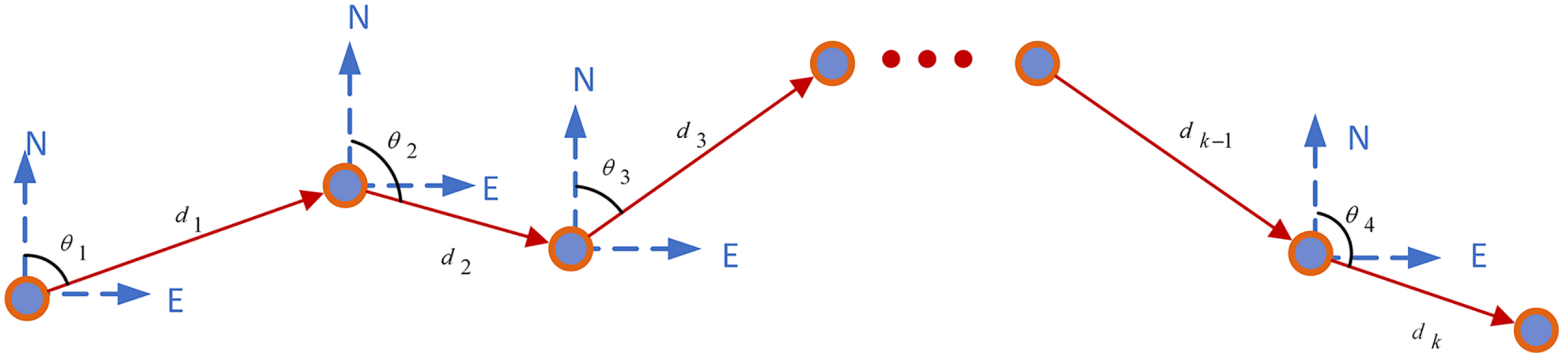
PDR principle block diagram.

The aim of PDR (Kemppi et al., 2010) positioning is to obtain the step length, step frequency, and heading of the pedestrian. For this, data are collected using an accelerometer, a gyroscope, and a magnetometer built into a smartphone carried by the pedestrian and used to calculate the pedestrian’s current position based on their position at a previous time. The PDR principle is illustrated using a block diagram in Fig. 2.

The predicted coordinate $\left( {{E_k},{N_k}} \right)$ can be calculated as follow:

(1) $$\left\{ {\matrix{ {{E_k} = {E_0} + \sum\limits_{n = 1}^k {{d_n}\sin {\theta _n}} } \cr {{N_k} = {N_0} + \sum\limits_{n = 1}^k {{d_n}\cos {\theta _n}} } \cr } } \right.$$

where the initial position is ${S_0}$, the coordinate is $\left( {{E_0},{N_0}} \right)$, ${d_n}$ indicates the step length, and ${\theta _n}$ indicates step heading angle.

#### Step detection and step length estimation

Human walking is a cyclic process, and acceleration sensor data in an accompanying smartphone also show cyclic variation. The walker’s pace is mainly calculated from periodic changes in acceleration information. Traditional pace detection algorithms include peak detection and zero-crossing (Yao et al., 2020). Peak detection accuracy based on the acceleration threshold is suboptimal because turning and sitting may be detected as a one-step change and lead to pseudo-wave peaks.

We set an acceleration threshold to filter out the sensor’s systematic error and a time threshold to exclude pseudo-wave peak. Then, under those conditions, the difference between the wave peak and the wave trough that reaches the threshold is determined and recorded as one step.

Step length estimation is used to estimate the length of each stride of the walker and is based on the statistical values of the walking status and sensor data. In PDR positioning systems, step length estimation errors are a primary source of cumulative errors. Moreover, step length is related to the individual pedestrian’s height, weight, gender, etc. (Susi, Renaudin & Lachapelle, 2013). A considerable volume of research is now focused on step length estimation. Step length is generally estimated using Eq. (2):

(2) $$d = {}^n\sqrt {{A_{\max }} - {A_{\min }}} \times C$$

where *n* = 4, (and can be modified according to different test conditions), ${A_{max}}$ is the maximum acceleration value in a stride cycle, ${A_{min}}$ is the minimum acceleration value in a stride cycle, and C is a constant.

#### Walking direction estimation

**Figure 3:**
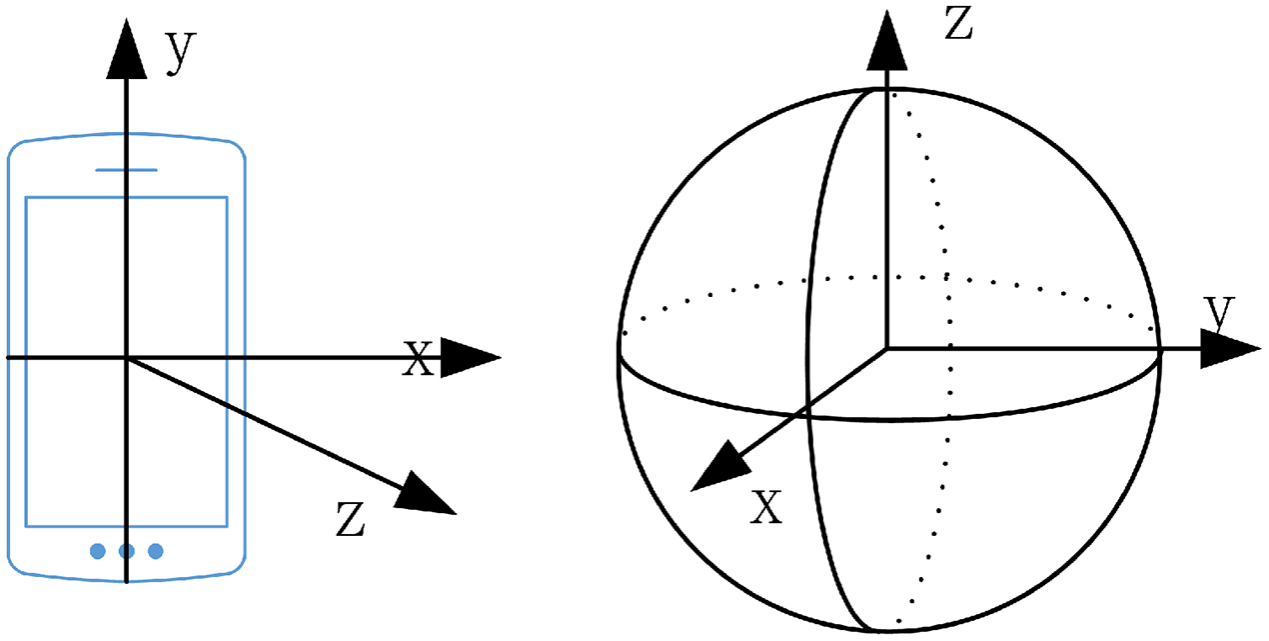
Schematic diagram of coordinate system (on the left is the mobile phone coordinate system, and on the right is the earth coordinate system).

The lecturer’s walking direction can be used as the basis for selecting the direction in which to rotate the camera and inferring the lecturer’s direction after they disappear from the camera’s view. The smartphone coordinate system is a relative coordinate system defined by the smartphone screen, as shown in Fig. 3. When the smartphone is placed horizontally, with the screen facing upward, the center of the cell phone screen is the coordinate origin (Zhang et al., 2016). The inertial coordinate system describes the state of motion in the objective world. It coincides with the phone coordinate system and is parallel to the axis of the world coordinate system. In our system, the phone coordinate system is transformed into the inertial coordinate system in a rotation operation. Figure 3 shows a Schematic diagram of the coordinate system (the left is the mobile phone coordinate system, and on the right is the earth coordinate system). Table 1 shows the sensor parameters.

**Table 1:** Sensor parameters.

Sensor type	Output data and illustration
Rotation-vector sensor	$value\left[ 0 \right]$, Rotation vector along the x-axis $\left[ {x \cdot \sin \left( {\displaystyle{\theta \over 2}} \right)} \right]$
	$value\left[ 1 \right]$, Rotation vector along the y-axis $\left[ {y \cdot \sin \left( {\displaystyle{\theta \over 2}} \right)} \right]$
	$value\left[ 2 \right]$, Rotation vector along the z-axis $\left[ {z \cdot \sin \left( {\displaystyle{\theta \over 2}} \right)} \right]$
	$value\left[ 3 \right]$, The value of the Rotation vector $\left[ {\cos \left( {\displaystyle{\theta \over 2}} \right)} \right]$

In addition to a gyroscope and an electronic compass that can determine direction, a smartphone also has a virtual rotation vector sensor. This is a software-based sensor that derives data from accelerometer, gyroscope and geomagnetic sensor data.

The output of the smartphone’s built-in rotation vector sensor is a standard quaternions. The rotation matrix around any axis of rotation can be constructed from the basis vectors. The rotation matrix is $R\left( {n,\theta } \right)$ is calculated as follows:

(3) $${}R\left( {n,\theta } \right) = \left[ {\matrix{ {n_x^2(1 - \cos \theta )}  {{n_x}{n_y}(1 - \cos \theta ) + {n_z}\sin \theta }  {{n_x}{n_z}(1 - \cos \theta ) - {n_y}\sin \theta } \cr {{n_x}{n_y}(1 - \cos \theta ) - {n_z}\sin \theta }  {n_y^2(1 - \cos \theta ) + \cos \theta }  {{n_y}{n_z}(1 - \cos \theta ) + {n_x}\sin \theta } \cr {{n_x}{n_z}(1 - \cos \theta ) + {n_y}\sin \theta }  {{n_y}{n_z}(1 - \cos \theta ) - {n_x}\sin \theta }  {n_z^2(1 - \cos \theta ) + \cos \theta } \cr } } \right]$$

Here, $n$ and $\theta$ represent the rotation matrix, $\lambda$, ${q_1}$, ${q_2}$ and ${q_3}$ are provided by $value\left[ 3 \right]$, $value\left[ 0 \right]$, $value\left[ 1 \right]$ and $value\left[ 2 \right]$, which are calculated using Eq. (4). The parameters of the rotation-vector sensor are shown in Table 1.

(4) $$\left\{ {\matrix{ {\lambda = \cos ({\theta \mathord{\left/ {\vphantom {\theta 2}} \right.} 2})} \cr {{q_1} = {n_x}\sin ({\theta \mathord{\left/ {\vphantom {\theta 2}} \right. } 2})} \cr {{q_2} = {n_y}\sin ({\theta \mathord{\left/ {\vphantom {\theta 2}} \right.} 2})} \cr {{q_3} = {n_z}\sin ({\theta \mathord{\left/ {\vphantom {\theta 2}} \right.} 2})} \cr } } \right.$$

From this rotation matrix, the attitude of the phone can be solved, the walking direction $\varphi$ can be calculated using Eq. (5), and the range of values of the heading angle is $\left[ {0,2\pi } \right]$.

(5) $$\varphi = \arctan \displaystyle{{2{q_1}{q_2} - 2{q_0}q{}_3} \over {1 - 2{q_1} - 2{q_3}}}$$

**Figure 4:**
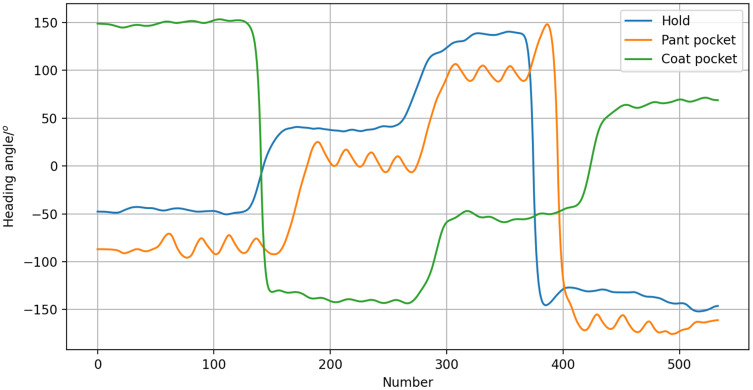
Heading angle of different carrying modes.

According to the heading angle, it is possible to determine the lecturer’s heading direction. Thus, when the lecturer disappears from the screen, the horizontal servo can determine whether to pan left or right based on the camera, and accurately rotate in the correct direction. Of course, the influence of how the smartphone is carried on the heading angle should be considered. We tested three carrying modes: the hold mode, the coat pocket mode, and the pant pocket mode. For this, a pedestrian carried a smartphone each way as they walked along a specified route. Changes in heading angle radian values were recorded to determine the impact of the carrying mode on the heading angle. As shown in Fig. 4, the different modes resulted in offset angles, but the trend was consistent. Therefore, it was concluded that the heading angle can still be used to estimate walking direction.

### Particle filter fusion of map information and camera angle

The particle filter algorithm is performed to fuse landmark point information and PDR information. The landmark point corresponding to the rotation angle of the camera is used as the observation data. We use the number of steps, step length, and direction of the speaker to model the lecturer’s behavior.

#### Particle filtering algorithm

Particle filtering (Djuric et al., 2003) is a non-parametric filtering algorithm, based on the Monte Carlo method, which can effectively deal with nonlinear systems. It can be applied to the target tracking field facing, which mainly solves the nonlinear and non-Gaussian problems. The update steps include particle initialization, particle weight update and random resampling.

#### Particle initialization

All particles are denoted as $H = \left\{ {X|i = 1,2,...,n} \right\}$, the state space of the $i$ particle is ${X^i} = {\left( {{x^i},{y^i},v_x^i,v_y^i} \right)^T}$, where the coordinates of particle $i$ is $\left( {{x^i},{y^i}} \right)$, $v_x^i$ and $v_y^i$ are the velocities of the particles in the $x$ and $y$ directions. The initial particle positions are $\left( {x_0^i,y_0^i} \right)$, which are Gaussian distribution. The position at k-moments is $\left( {{x_t},{y_{t - 1}}} \right)$. The duration of $t$ and $t - 1$ is $\Delta{t_k}$. The step length is ${d_s}$, the angle $\theta$ is between motion direction and $y$-axis. The position of the lecturer at time $t$ is $\left( {{x_t},{y_t}} \right)$ as follow:

(6) $$\left\{ {\matrix{ {{x_t} = {x_{t - 1}} + {d_s}\sin \theta } \cr {{y_t} = {y_{t - 1}} + {d_s}\cos \theta } \cr } } \right.$$

The speed of walking does not vary much when a person is walking normally, which has the same speed at moment $t - 1$ and moment $t$. The speed of walking can be calculated as follow:

(7) $$\left\{ {\matrix{ {{v_{x,t}} = {v_{x,t - 1}}} \cr {{v_{y,t}} = {v_{y,t - 1}}} \cr } } \right.$$

where ${v_{x,t}}$ and ${v_{y,t}}$ indicate the speed along x-axis and y-axis.

(8) $${\rm w:}\left[ {\matrix{ {{x_t}} \cr {{y_t}} \cr {{v_{x,t}}} \cr {{v_{y,t}}} \cr } } \right] = \left[ {\matrix{ {{x_{t - 1}}} \cr {{y_{t - 1}}} \cr {{v_{x,t - 1}}} \cr {{v_{y,t - 1}}} \cr } } \right] + \left[ {\matrix{ {{d_s}\sin \theta } \cr {{d_s}\cos \theta } \cr 0 \cr 0 \cr } } \right] + \left[ {\matrix{ {{\sigma _c}} \cr {{\sigma _c}} \cr {{\sigma _v}} \cr {{\sigma _v}} \cr } } \right]$$

#### Particle weight update

Particle weight is updated when a new observation value (the corresponding angle of the PT camera) is obtained at the time. Then the weights are updated according to the observations. After the weight is updated, a new selection is made for the particles. The rule is to discard the particles with smaller weight and retain the particles with larger weight. The new particle weight value adopts Gaussian distribution, the observation value is the initial position, and the distance between each particle and the observation value is the European distance. We take this distance as the standard deviation; its weight calculated as follows:

(9) $$w_t^i = p\left( {{z_t}|x_t^i} \right) = \displaystyle{1 \over {\sqrt {2\pi } \varsigma }}\exp \left[ { - \displaystyle{{{{\left( {d_t^i} \right)}^2}} \over {2{\varsigma ^2}}}} \right]$$

where $d_t^i$ is the distance between the particle and the observation position $\left( {x_t^m,y_t^m} \right)$, $\varsigma$ is the standard deviation. The $d_t^i$ is calculate as:

(10) $$d_t^i = \sqrt {{{\left( {x_t^i - x_t^m} \right)}^2} + {{\left( {y_t^i - y_t^m} \right)}^2}}$$

The reference landmark determined by the current angle of the PT camera and the face position detected on the screen. The reference set points in the system mainly include:

1. When the current horizontal angle of the PT is ${90^ \circ }$, the vertical angle is ${45^ \circ }$, and the face is in the middle, the corresponding coordinates $\left( {0,0} \right)$;
2. When the current horizontal angle of the PT is ${130^ \circ }$, the vertical angle is ${45^ \circ }$, and the face is in the middle, the corresponding coordinates $\left( {2,0} \right)$;
3. When the current horizontal angle of the PT is ${50^ \circ }$, the vertical angle is ${45^ \circ }$, and the face is in the middle, the corresponding coordinates $\left( { - 2,0} \right)$;

The particle weights are affected by the motion model, where $w_t^{{\rm *}i}$ is then normalized to get the new weights as follow:

(11) $$w_t^{ * i} = w_t^i/\sum\limits_{i = 1}^{{N_s}} {w_t^i}$$

where ${N_s}$ is the number of particles, ${N_s}$ is set to 100 in our proposed particle filtering. Several studies have shown that, the higher the number of particles, the higher the accuracy of the localization, but the time consumed will also be longer, which conflicts with the requirement of real-time. Therefore, in our article, ${N_s}$ is set to 100 in our proposed algorithm. After the particle state and the particle weight updated, the updated state of the system will be generated. The current state of systems ${X_t} = {\left( {{x_t},{y_t},{v_{x,t}},{v_{y,t}}} \right)^T}$ is represented as follow:

(12) $${X_t} = \sum\limits_{i = 1}^n {w_t^{ * i}x_t^i}$$

The position of the current lecturer is $\left( {{x_t},{y_t}} \right)$. The position obtained by the PDR system can be used as a supplement to the absence of facial information in the face information. We convert the position into the angle that the PT camera needs to rotate.

#### Map-aided particle filter

Particle filtering fusion of head rotation angle and PDR information is used to solve the localization problem. Nevertheless, there is a problem of predicted trajectory through the wall in the actual experiment because the podium is modeled. A floor map records the positions of the walls in an indoor scenario, and the walls are not to be passed through. Accordingly, when the particles are detected to pass through the walls, the particle weights are updated using Eq. (13):

(13) $${w_c} = \left\{ \matrix{
  0\quad cross\;the\;wall \hfill \cr 
  1\quad \quad otherwise \hfill \cr}  \right.$$

According to the Eq. (13), corresponding weight of the particle filter will be set to zero, when the predicted position of a particle is outside the valid region of the map. The new particle weights $w_t^i$ is updated as follow:

(14) $$w_t^i = {w_c}w_t^{ * i}$$

Based on this new particle weight, the particles will be normalization and resampling.

### Components of proposed system

**Figure 5:**
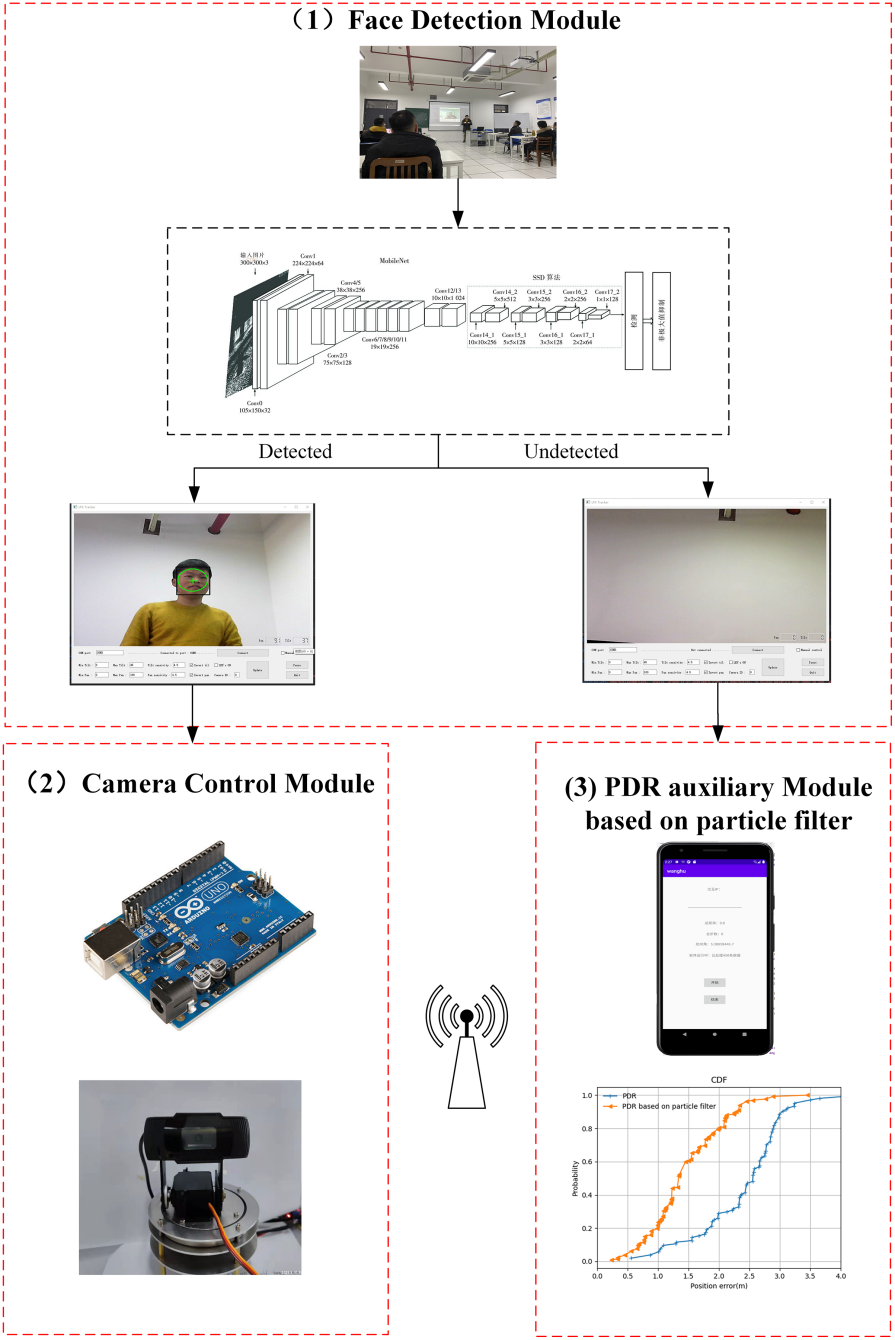
The framework of the proposed system.

In this section, all the components of the proposed system are described. There are three modules: (a) face detection module, (b) camera control module, and (c) PDR auxiliary module based on particle filtering. First, face detection is employed to detect the lecturer’s face. Next, the PDR auxiliary module is used to locate the lecturer as they are moving. The lecturer’s location information is transmitted to the server through Wi-Fi. The camera rotation control module has two control strategies: the rotation angle is calculated based on the offset of the picture and the offset of the position information. The framework of the proposed system is shown in Fig. 5.

### Face detection module

In recent years, face detection has been widely adopted in computer vision. In the lecturer tracking process, the main function of this module is to detect the face. Considering that our proposed system has edge-based detection, it was deemed suitable to adopt MobileNet. In addition, the Intel OpenVINO toolkit optimizer (Kustikova et al., 2019) has been applied to enhance face detection efficiency.

#### Backbone

**Figure 6:**
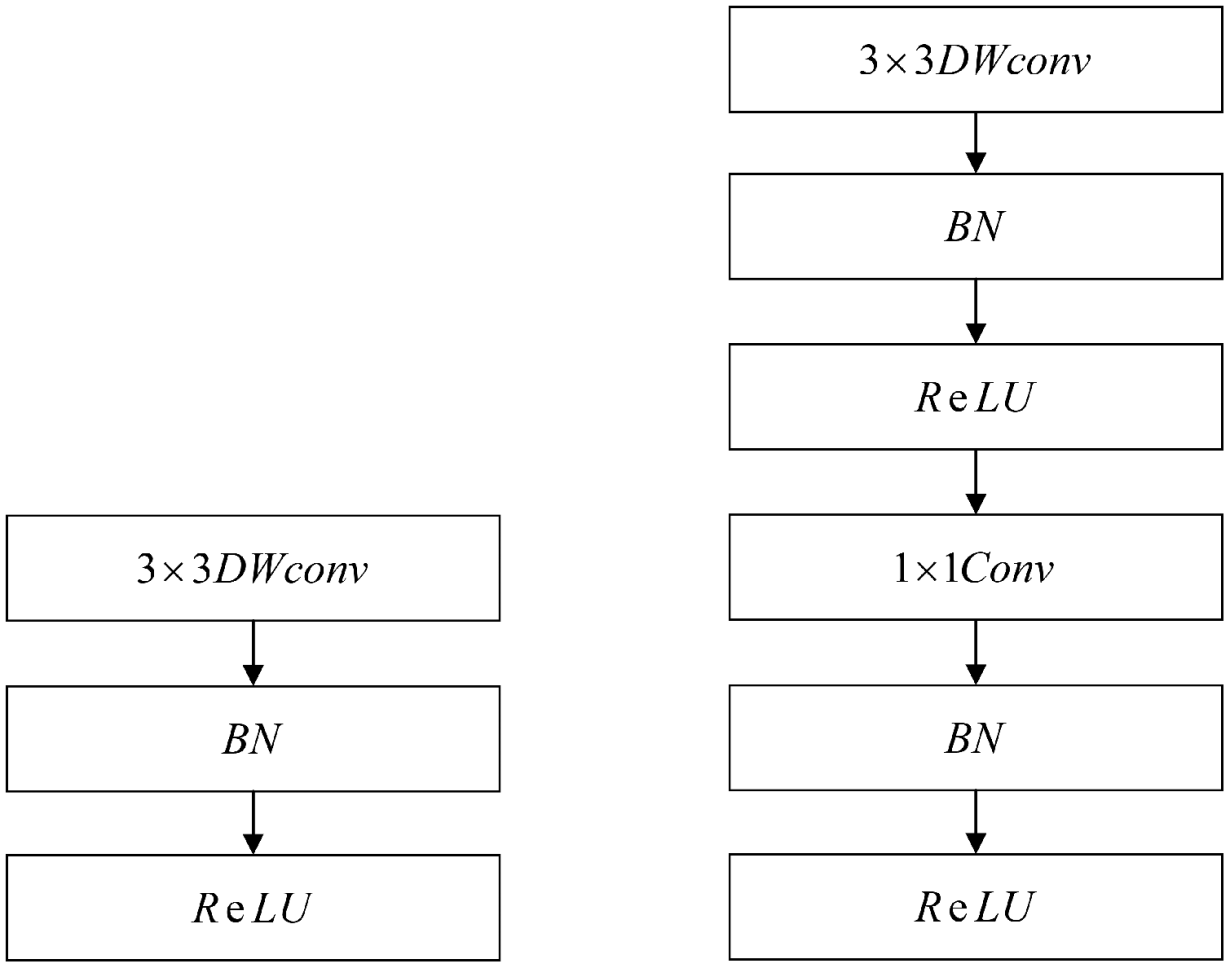
Network implementation of the MobileNet.

The face detection module is based on the MobileNet-SSD framework. MobileNets (Howard et al., 2017) is an efficient framework that was proposed by Google in 2017. It deploys a low-latency model through two collaterals suitable for mobile edge devices. It is a single-stage detection method that mainly uses depth-separable convolution to decompose the standard convolution kernel. At the same time, two hyperparameters were introduced to reduce the number of parameters and the computational workload. In addition, MobileNet-SSD has two important features. First, it uses MobileNet to replace the VGG-16 background network. Second, the depth convolution in MobileNet separates the standard 3*3 convolution, turning it into 3*3 depth separable convolution and 1*1 point convolution. Each convolution layer is followed by a batch normalization layer and Rectified Linear Units (ReLU) activation function layer; the network implementation is shown in Fig. 6.

#### OpenVINO

**Figure 7:**
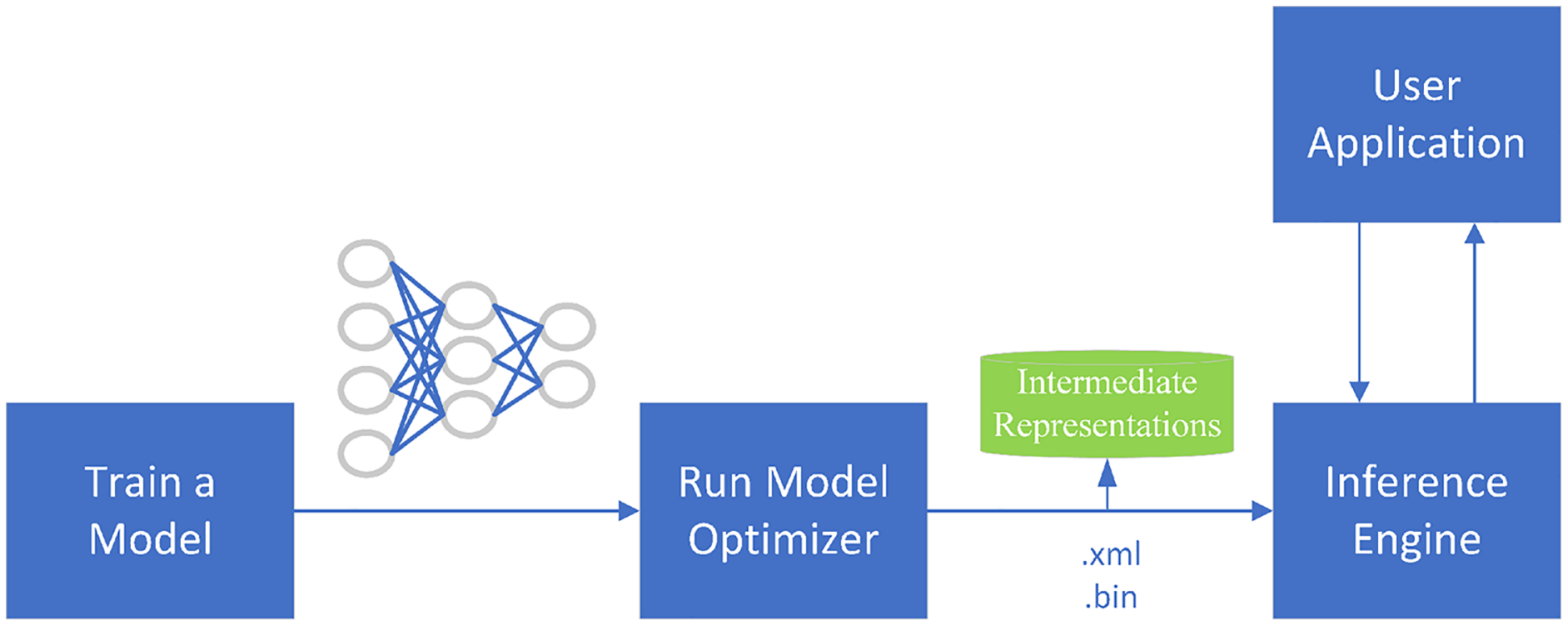
OpenVINO optimization process.

OpenVINO is a toolkit for the rapid development of applications and solutions that Intel developed based on its hardware platforms. The toolkit accelerates and optimizes the legacy APIs of OpenCV, OpenXV vision libraries to run on CPU, GPU, and FPGA. The toolkit is based on the latest generations of artificial neural networks, including CNNs. As shown in Fig. 7, the main components of the toolkit are model-optimizer and an inference engine. The model optimizer is a cross-platform command-line tool that converts trained neural networks from their source frameworks into intermediate representations for inference operations. The inference engine supports the accelerated operation of deep learning models at the hardware instruction set level, as well as the instruction set optimization of the traditional OpenCV image processing library.

We choose the face-detection-adas-0001 network based on the MobileNet-SSD framework. This model is used in driver monitoring and similar scenarios. It has been optimized on the Caffe framework using the OpenVINO toolkit, which can run at 25 frames per second on the CPU. We also compared it with other model frameworks provided by OpenVINO and finally selected it for experimental testing. As shown in Table 2, the deep learning evaluation index Average Precision (AP) was introduced to evaluate these models. It was found that face-detection-adas-0001 had the highest AP.

**Table 2:** Comparison of detection accuracy.

Model	Shape for input	Backbone	AP (%)
Face-detection-adas-0001	$\left[ {1 \times 3 \times 384 \times 672} \right]$	Mobilenet	94.1
Face-detection-adas-binary-0001	$\left[ {1 \times 3 \times 384 \times 672} \right]$	Mobilenet	91.9
Face-detection-retail-0001	$\left[ {1 \times 3 \times 300 \times 300} \right]$	SqueezeNet-light+ssd	83

### Camera control module

Arduino UNO R3 is a crucial component of the proposed camera control module. This module contains the microcontroller ATmega328 and a PT camera. It directs the camera to perform horizontal and tilt movements, extends the monitoring range, and ensures that the lecturer is always within the camera’s field of view. In addition, a reasonable PT control strategy should minimize unnecessary jitter. That is, the PT movement should be smooth. The face detection module acquires the tracking target to guide the movement of the PT camera.

#### Pan-Tilt camera control strategy

**Figure 8:**
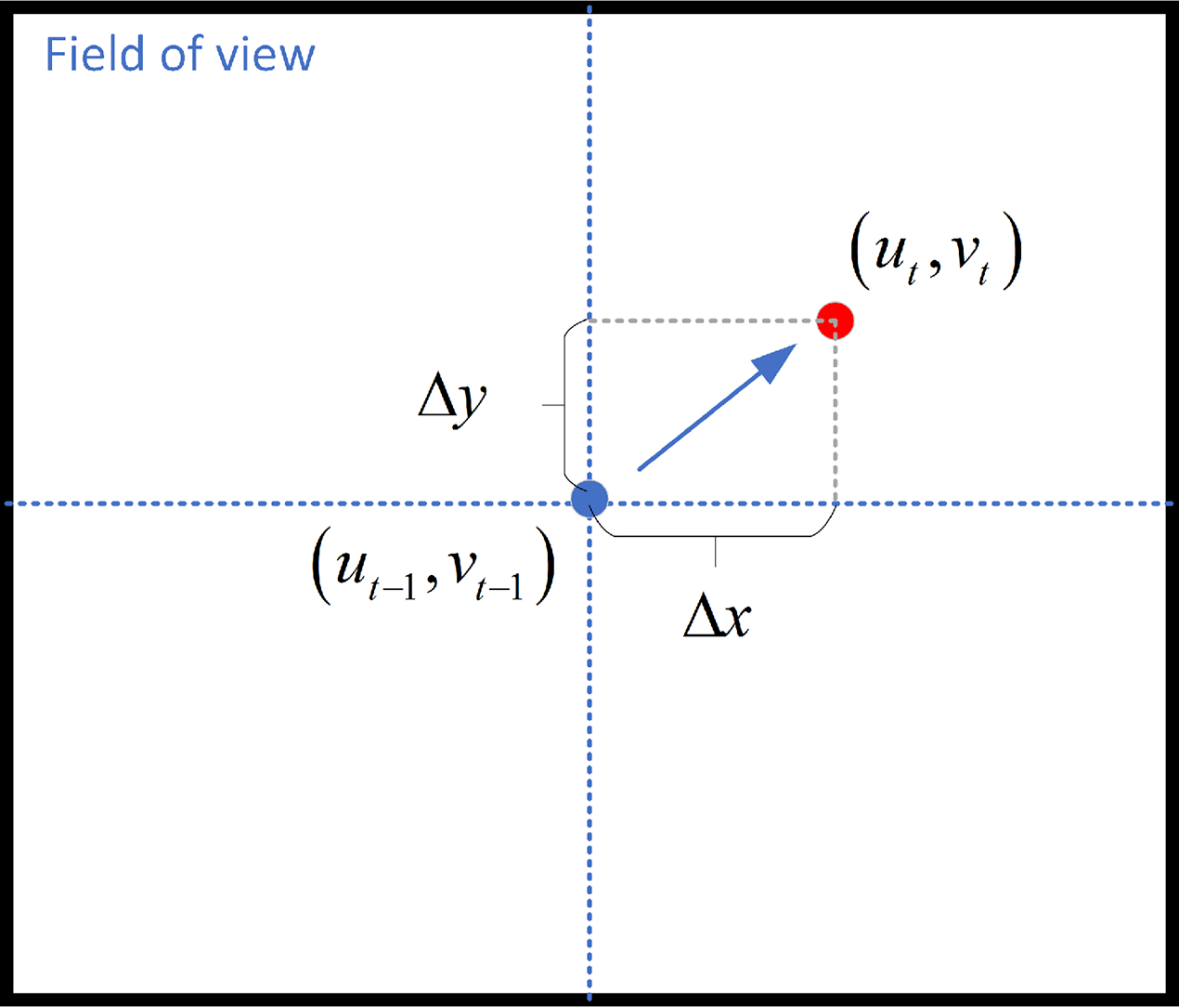
Camera’s field of view.

As stated above, we have established the coordinate system related to the PT camera. When the ${t^{th}}$ frame is set, the target tracking algorithm calculates and outputs the pixel coordinates of the center image of the target frame as $\left( {{u_t},{v_t}} \right)$, and the target position in the previous frame is $\left( {{u_{t - 1}},{v_{t - 1}}} \right)$, Fig. 8 shows the camera’s field of view.

We control the rotation of the PT camera by computing the distance between the face and the center of the screen. The face detection module draws the bounding box of the detected face and calculates the offset angle based on the distance between the bounding box and the center. The formula for calculating the offset angle as follow:

(15) $$\left[ {\matrix{ {\Delta{\theta _x}} \cr {\Delta{\theta _y}} \cr } } \right] = \left[ {\matrix{ {\arctan \displaystyle{{\Delta x} \over f}} \cr {\arctan \displaystyle{{\Delta y} \over f}} \cr } } \right] = \left[ {\matrix{ {\arctan \displaystyle{{\left( {{u_t} - {u_0}} \right)dx} \over f}} \cr {\arctan \displaystyle{{\left( {{v_t} - {v_0}} \right)dy} \over f}} \cr } } \right]$$

where $\Delta{\theta _x},\Delta{\theta _y}$ are the offset angle along the x-axis and y-axis, and $dx$ and $dy$ are the physical dimensions of each pixel in the $X$-axis and $Y$-axis, $\left( {{u_0},{v_0}} \right)$ is the center point of the screen, $f$ is the focal length. The focal length of the camera is 2.8 mm.

#### Tracking strategy when the target disappears

**Figure 9:**
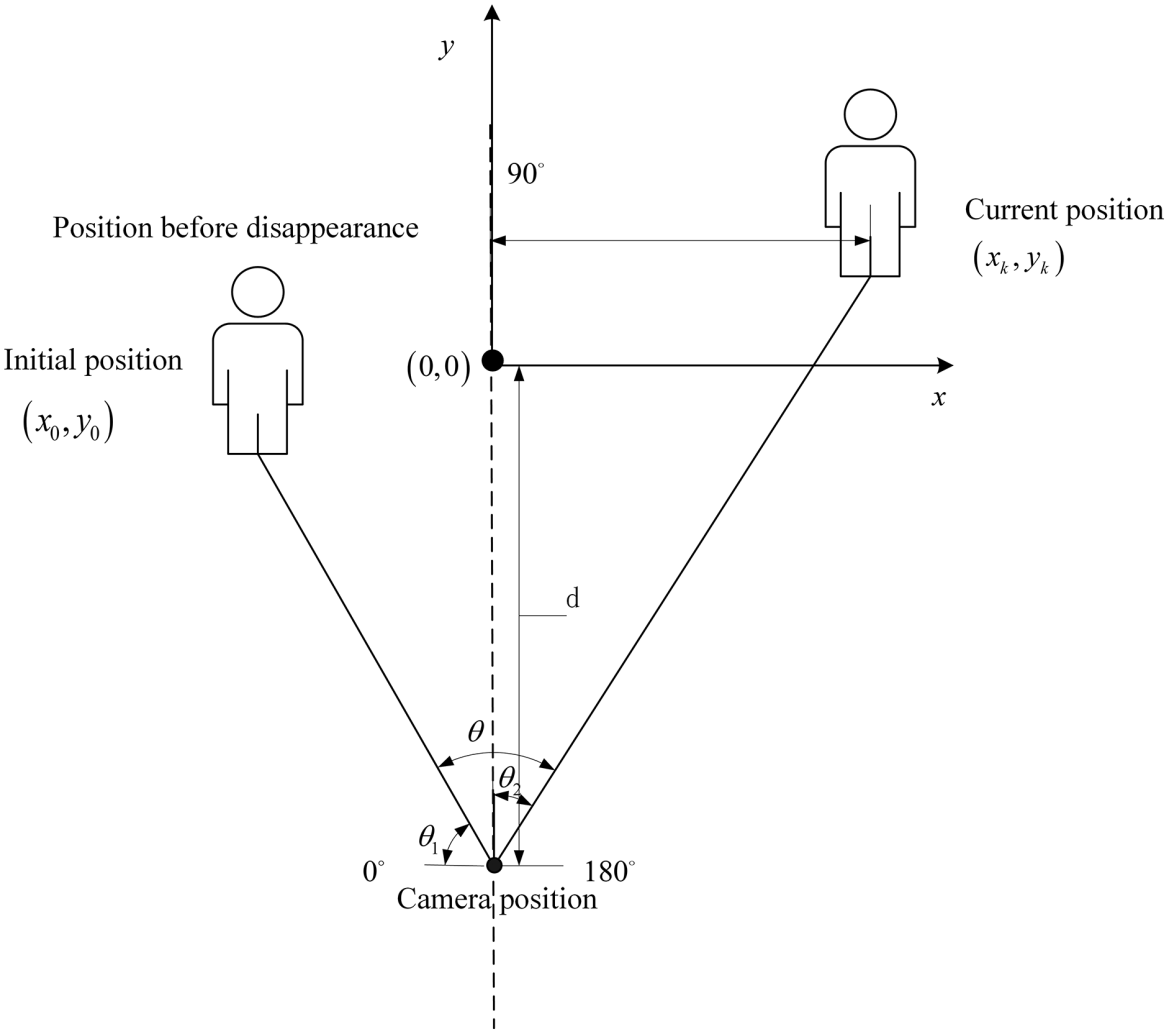
Control strategy of the PT camera.

When the target disappears from the camera’s view, the PDR module uses the coordinates of the lecturer’s position collected by their smartphone to locate the lecturer and calculate the rotation angle. The PT camera is then rotated. As shown in Fig. 9, the offset angle is calculated based on the lecturer’s current position.

The angle of the PT camera is calculated from the position in the real environment, and ${\theta _2}$ is the rotation angle from an initial position, calculated as follows:

(16) $${\theta _2} = \arctan \left( {\displaystyle{{{x_k}} \over {d + y{}_k}}} \right)$$

(17) $$\theta = \left\{ {\matrix{ {\displaystyle{\pi \over 2} + {\theta _2}{\rm }\Big(0 \lt {\theta _1} \lt \displaystyle{\pi \over 2}\Big)} \cr {\displaystyle{\pi \over 2} - {\theta _2}{\rm }\Big(\displaystyle{\pi \over 2} \lt {\theta _1} \lt \pi \Big)} \cr } } \right.$$

where the $\left( {{x_k},{y_k}} \right)$ is the current position calculated from PDR module, $d$ is the distance between the lecturer’s initial position and the PT camera, and $\theta$ is the rotation angle.

### PDR auxiliary module based on particle filtering

When the lecturer’s motion trajectory is smooth, the face detection module can be utilized. Nevertheless, the face detection module will return accurate results when the lecturer moves suddenly or swiftly. This is a universal problem in learning-based tracking methods (Huang, Li & Nevatia, 2013). To overcome this obstacle, we have introduced a PDR-based method for when face detection method is not feasible. At the same time, a particle filter algorithm is applied to further integrate the obtained position information. This can help the system detect the lecturer when they are out of the camera’s field of view and determine their position in the two-dimensional plane. The motion blur and scale-variance caused by rapid movement can likewise be resolved to a certain extent through this operation. The Android mobile application developed in Android studio can obtain PDR information. Through the particle filter deployed on the server, the pedestrian trajectory estimation information is fused with the absolute positioning information provided by the PT camera.

### Experimental evaluation

We conducted various experiments to evaluate the performance of the intelligent lecture recording system and to compare it to alternative approaches. Here, the findings are presented, as well as a brief discussion of the impact of the particle filter on positioning error and the advantages and disadvantages of several related systems.

### Experimental environment

**Figure 10:**
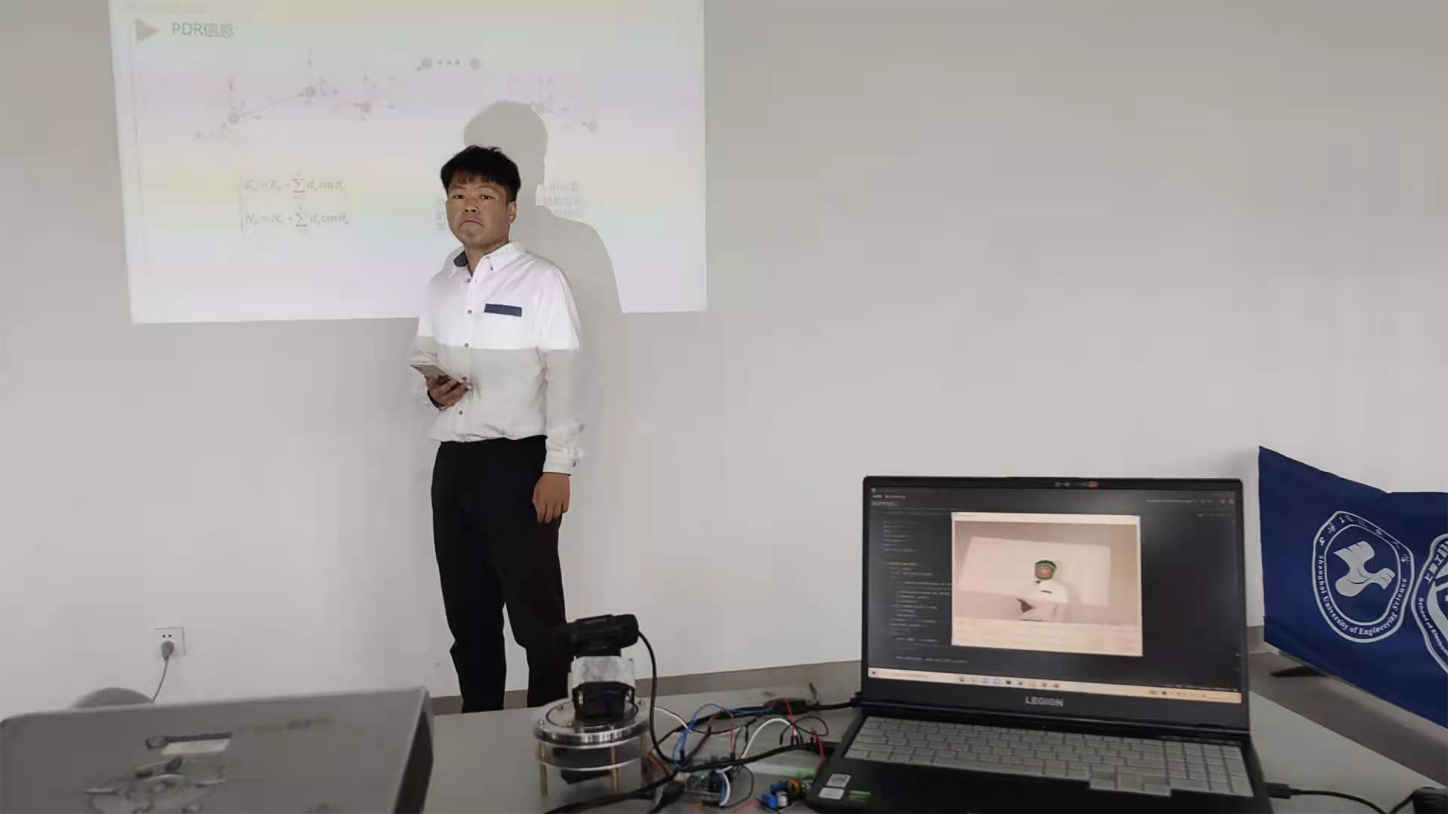
Experimental environment.

Experiments in this research are carried out in the classroom, laboratory, and conference room respectively. The computing platform is under Python 3.7 with a 2.8 GHz Intel i7-10750H and 16G RAM. The resolution of the videos is 384*672, and the video is 25 FPS. Figure 10 gives an example of the experimental environment.

**Figure 11:**
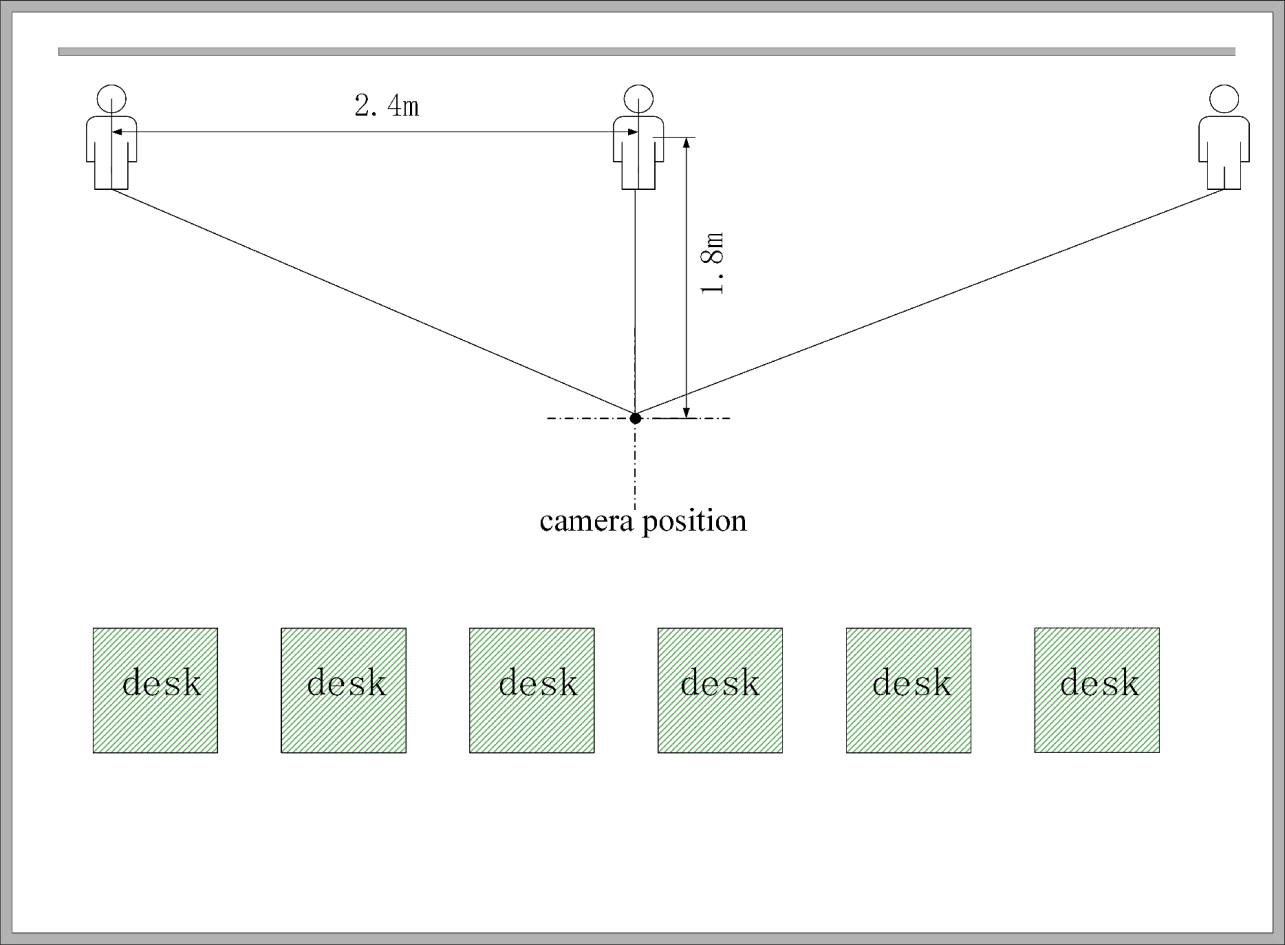
Schematic diagram of the experimental scene.

The experimental results are presented in the context of two distances used to determine the range of the PT camera’s rotation. As depicted in Fig. 11, the two distances were (1) the distance between the PT camera and the lecturer and (2) the range of the lecturer’s movement. The important distances are shown in Table 3.

**Table 3:** The important distance.

Scenario	The distance between the PT camera and the lecturer (m)	The range of the lecturer’s movement (m)
Conference room	1.2	3.0
Laboratory	1.5	4.2
classroom	1.8	4.8

**Figure 12:**
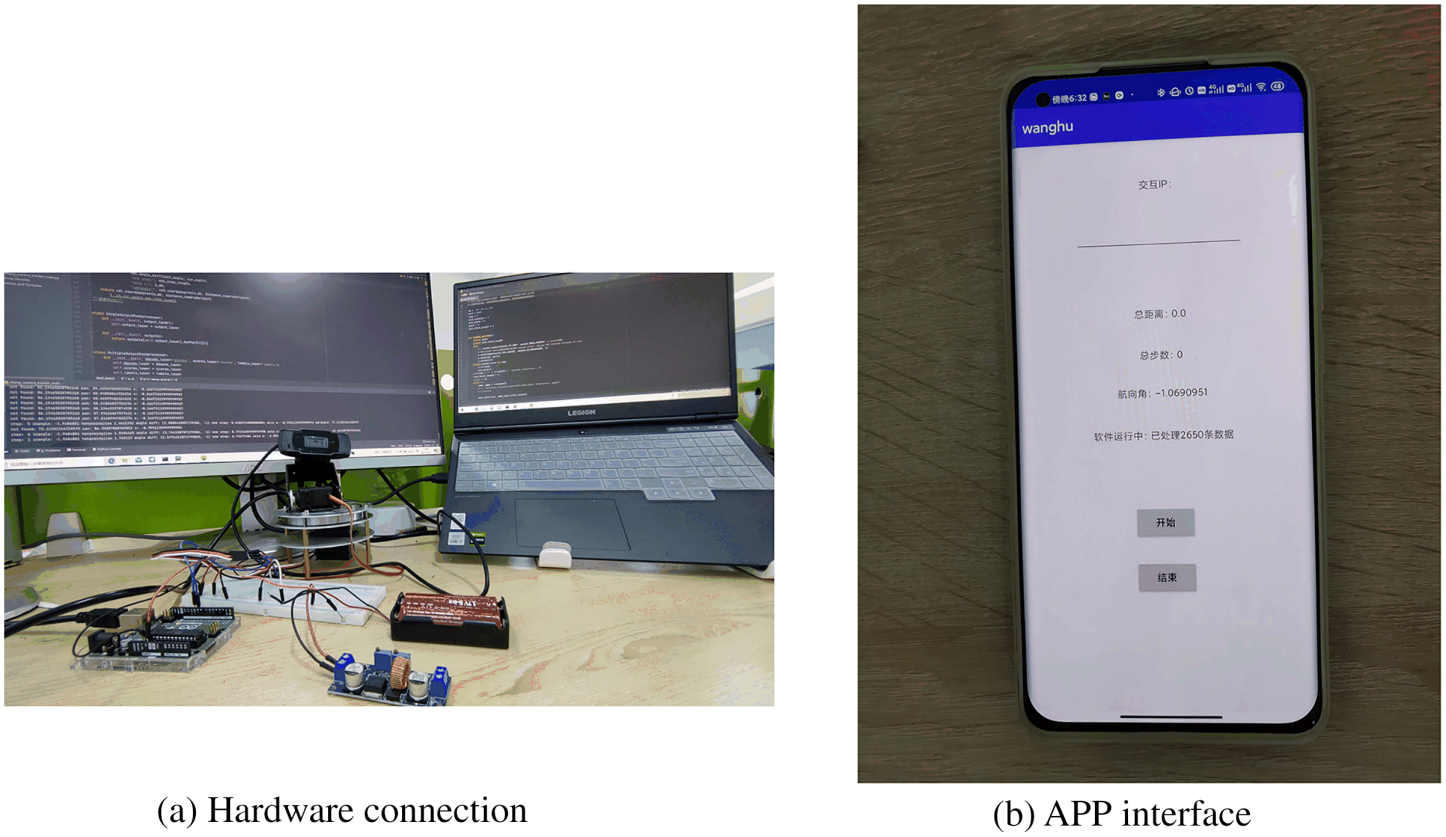
Devices used in experiments. (A) Hardware connection diagram. (B) App interface.

In this system, the transmission of PDR data is accomplished by establishing a Socket TCP/IP communication with the lecturer’s smartphone. A laptop is used as the server, and the mobile app is set as the client. Furthermore, serial communication is established between the Arduino controller and the PT camera. The application runs on a Xiaomi 11 smartphone with a sampling frequency of 50 HZ. The smartphone obtains the required data by accessing the built-in API interface of Android Studio. As shown in Fig. 12, the laptop, as the server, communicates with Arduino UNO R3 through the serial port. The smartphone uses sensors to collect data and then transmits the data to the laptop for processing *via* Wi-Fi to satisfy real-time tracking requirements. Figure 12A shows the hardware connection, including the laptop, PT camera, and control system based on Arduino. Figure 12B shows the mobile app page that displays PDR information. The hardware and software platforms employed in the system are shown in Table 4.

**Table 4:** Hardware and software platforms.

Hardware	Software
Arduino uno R3	Arduino IDE 1.8.13 PyCharm in python3.7 Android studio
Servo moto HWZ020	
Philips web-camera	
MI 11 phone	
Adjustable voltage regulator chip	
Power supply battery	

### Evaluation metrics

To evaluate the performance of the system, we introduced two evaluation metrics. The first is the $Center\_rate$. It can be calculated by the Eq. (18).

(18) $$Center\_rate = \displaystyle{{Center\_num} \over {Frame\_num}}$$

where $Center\_num$ is the number of the frames when the lecturer appears in the center of the camera’s field of view. $Frame\_num$ is the number of all frames in the video. In order to avoid unnecessary shaking, we set a dead zone which is an offset less than 100 pixels from the screen. The camera will not rotate within this zone. It indicates that the lecturer is in the center of the screen. When the lecturer moves frequently, the lecturer is still in the camera frame. Therefore, we considered another ratio $In\_rate$. It can be calculated by the Eq. (19)

(19) $$In\_rate = \displaystyle{{In\_num} \over {Frame\_num}}$$

where $In\_num$ represents the number of the frames when the lecturer appears in the camera’s field of view.

In addition, to evaluate the system’s robustness, the duration and frequency of invisible speakers in different videos are determined as another dimension of evaluation. The duration is the time that the speaker vanishes from the camera. Here we use slight and severe errors to illustrate the system’s robustness.

1. Slight error: The PT camera performs an incorrect operation, but it recovers immediately within a few seconds (Error time <= 10 s);
2. Severe error: The PT camera performs a wrong move and does not return to the lecturer for a longer time (Error time > 10 s).

### Results in different scenarios

The evaluation experiments were conducted in three different scenarios: a conference room, a laboratory, and a classroom. Table 5 lists the percentages of the proposed metrics for the 18 videos recorded in these scenarios. The experiments described in this section were performed with the smartphone in hold mode.

**Table 5:** Results in three different scenarios.

	$Frame\_num$	$Center\_num$	$In\_num$	$Center\_rate$ $\left( \% \right)$	$In\_rate$ $\left( \% \right)$
Video	Recorded in the conference room				
Video1	1,355	920	1,250	67.89	92.25
Video2	1,840	1,160	1,625	63.03	88.31
Video3	2,320	1,525	2,105	65.72	90.73
Video4	1,785	1,150	1,595	64.43	89.36
Video5	2,585	1,710	2,320	66.15	89.75
Video6	2,465	1,595	2,230	64.71	90.47
Average	2,058	1,343	1,854	65.32	90.15
Video	Recorded in the laboratory				
Video7	1,300	810	1,150	62.30	88.46
Video8	2,390	1,525	2,035	63.81	85.15
Video9	2,030	1,245	1,775	61.33	87.44
Video10	1,985	1,090	1,670	54.91	84.13
Video11	2,230	1,255	1,905	56.28	85.43
Video12	1,920	1,135	1,685	59.11	87.76
Average	1,976	1,176	1,703	59.62	86.40
Video	Recorded in the classroom				
Video13	1,200	785	1,095	65.42	91.25
Video14	1,345	870	1,235	64.68	91.82
Video15	2,175	1,285	1,875	59.08	86.21
Video16	2,670	1,665	2,435	62.36	91.20
Video17	2,370	1,435	2,075	60.55	87.55
Video18	1,875	1,130	1,660	60.27	88.53
average	1,939	1,195	1,729	62.06	89.42

As indicated in Table 5, $Center\_rate$ of the videos captured in the laboratory varied from 54.80% to 63.89%, and the $In\_rate$ varied from 84.13% to 88.46%. The average $Center\_rate$ of the videos recorded in the classroom was 2.44% higher than that of the video recorded in the laboratory, and the average $In\_rate$ was 3.02% higher. The best performance metrics were obtained in the conference room. The $Center\_rate$ in the conference room ranged from 64.43% to 67.89%, and the $In\_rate$ varied from 88.31% to 92.25%. To determine why the performance varied in these different scenarios, we further analyzed the impact of the distance between the camera and the lecturer.

**Figure 13:**
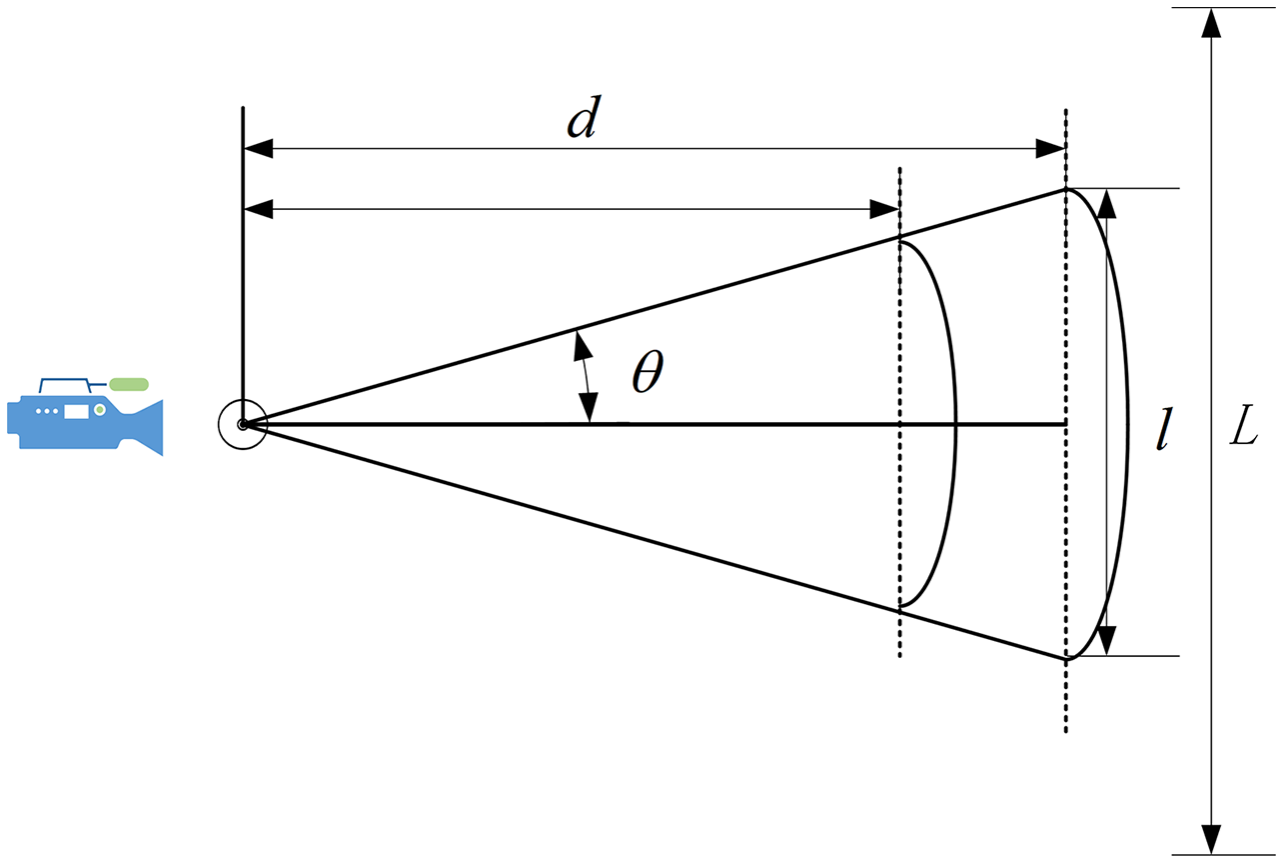
Distance analysis.

As shown in Fig. 13, where $d$ is the distance between the camera and the Podium, $\theta$ is the camera’s angle in the field of view, $l$ is the horizontal field of view range, and *L* is the range of the lecturer’s movement. The field of view can be calculated based on the distance and offset angle in this system. Triangle basis knowledge can be used to calculate $l$ as follows:

(20) $$l = 2d\tan \theta$$

From Table 6, we know that as *d* increases, the corresponding field of view will also increase. In these scenarios, a small camera rotation angle allows the camera to cover a large range. Therefore, in the classroom—which had a larger *l*—we obtained better results than in the laboratory. In addition, the value of *d* was slightly reduced in the conference room, but the range of the lecturer’s movement *L* was significantly reduced. In this case, the camera did not need to rotate frequently. At the same time, the lecturer’s walking speed was limited, and there was no sudden movement. Therefore, the scenario in the conference room also returned good results, even though it had the shortest distance between the camera and the podium. In our system, the face detection module requires a minimum head size of 90 × 90 pixels, so *d* should ideally be 1.6–2.0 m for our system to provide satisfactory results.

**Table 6:** The horizontal field of view range.

The field of view	${d}$ (m)	${l}$ (m)	${\theta }$ ( $^\circ$)	*L* (m)
Conference room	1.2	1.330	29	3.0
Laboratory	1.5	1.662	29	4.2
Classroom	1.8	1.996	29	4.8

Table 7 shows the error counts in three scenarios, the overall runtime of the recordings, the number of error instances, and the total duration of errors. We found that slight and severe errors were rare in our proposed system. The occasional error was caused by the unsmooth rotation of the PT camera.

**Table 7:** Error in three scenarios.

Scenario	Runtime (min)	Slight error count	Severe error count	Duration (s)
Conference room	10	4	2	42
Classroom	10	4	2	50
Laboratory	10	5	3	62

### Results in different carrying mode of the smartphone

We also considered the impact of the smartphone carrying mode on the system’s performance. The smartphone was carried either in the lecturer’s coat pocket or pant pocket, and the video was recorded in the classroom scenario. Table 8 shows the system’s performance when the smartphone was in the coat pocket and in the pant pocket, and these results can be compared with the results of the hold mode presented in Table 5.

**Table 8:** Impact of carrying mode on the system.

	$Frame\_num$	$Center\_num$	$In\_num$	$Center\_rate$ $\left( \% \right)$	$In\_rate$ $\left( \% \right)$
Video	Recorded in the coat pocket mode				
Video1	1,420	850	1,210	59.86	85.21
Video2	1,990	1,195	1,675	60.05	84.17
Video3	2,050	1,205	1,725	58.78	84.15
Video4	1,750	1,075	1,515	61.43	86.57
Video5	2,155	1,275	1,855	59.16	86.07
Video6	2,415	1,455	2,100	60.25	86.96
Average	1,963	1,175	1,680	59.92	85.52
Video	Recorded in the pant pocket mode				
Video7	1,300	765	1,095	58.85	84.23
Video8	1,645	970	1,405	58.97	85.41
Video9	2,055	1,195	1,760	58.15	85.64
Video10	2,470	1,405	2,035	56.88	82.39
Video11	2,320	1,335	1,975	57.30	84.76
Video12	1,875	1,060	1,570	56.53	83.73
Average	1,944	1,121	1,640	57.78	84.36

The findings indicate that the proposed system’s tracking function is still reasonable when the smartphone is in a coat or pant pocket. The $\; Center\_rate$ of the videos recorded with the smartphone in the coat pocket was 58.78–60.25%, and the $In\_rate$ varied from 84.15% to 86.96%. The quality of the videos recorded when the smartphone was in the pant pocket or coat pocket was similar; however, the heading angle fluctuated to a certain extent due to the shaking of the smartphone in the pant pocket. The metrics were slightly lower for both modes compared to the hold mode. However, good tracking could still be achieved. Therefore, it can be concluded that the smartphone carrying mode has a small impact on the system’s performance and that the hold mode, like a fixed IMU, delivers the best performance. Although it should be noted that, from a cost and complexity perspective, adding a fixed IMU creates additional system costs—there is a cost–performance trade-off.

### The impact of the PDR auxiliary module on the system

To evaluate the impact of the PDR auxiliary module, we used only the face detection module to record videos without PDR information. Table 9 shows the metrics for the classroom scenario videos recorded without the PDR auxiliary module. As indicated in Table 9, the performance of the system without the PDR auxiliary module was considerably reduced. The average $Center\_rate$ was 15.05% lower, and the $In\_rate$ was about 30% lower.

**Table 9:** Videos without PDR auxiliary module.

Video	System without PDR auxiliary system in classroom				
	$Frame\_num$	$Center\_num$	$In\_num$	$Center\_rate\ \left( \% \right)$	$In\_rate\ \left( \% \right)$
Video1	1,300	635	785	48.89	60.39
Viedo2	1,890	860	1,100	45.87	58.20
Video3	2,080	990	1,240	49.09	59.62
Video4	2,445	1,155	1,425	45.67	58.28
Video5	1,875	875	1,130	47.08	60.27
Video6	2,075	945	1,235	45.48	59.52
Average	1,944	910	1,152	47.01	59.38

As shown in Table 10, the count of slight and severe errors in the system without the PDR auxiliary module was significantly higher than in the proposed system, and the error duration time increased considerably. Clearly, the PDR auxiliary module significantly improves the proposed system’s performance.

**Table 10:** The comparison of error count.

Error count	Runtime (min)	Slight error count	Severe error count	Duration (s)
Proposed system	10	4	2	50
System without PDR module	10	8	6	142

### Particle filter positioning error analysis

Here, we used the average positioning error and the cumulative distribution probability of the positioning error as measurement metrics for positioning performance. The cumulative distribution probability of the positioning error is the cumulative probability that the positioning error is less than a specific value. In this experiment, the lecturer walked on a classroom stage holding a smartphone while the system recorded. In several test cases, the lecturer walked along the same path several times. Both the actual trajectory and the predicted trajectory were recorded. These experimental cases were divided into two groups: one group used PDR positioning without a particle filter, and one group used PDR based on the particle filter algorithm. All positioning errors were counted according to the mean values.

**Figure 14:**
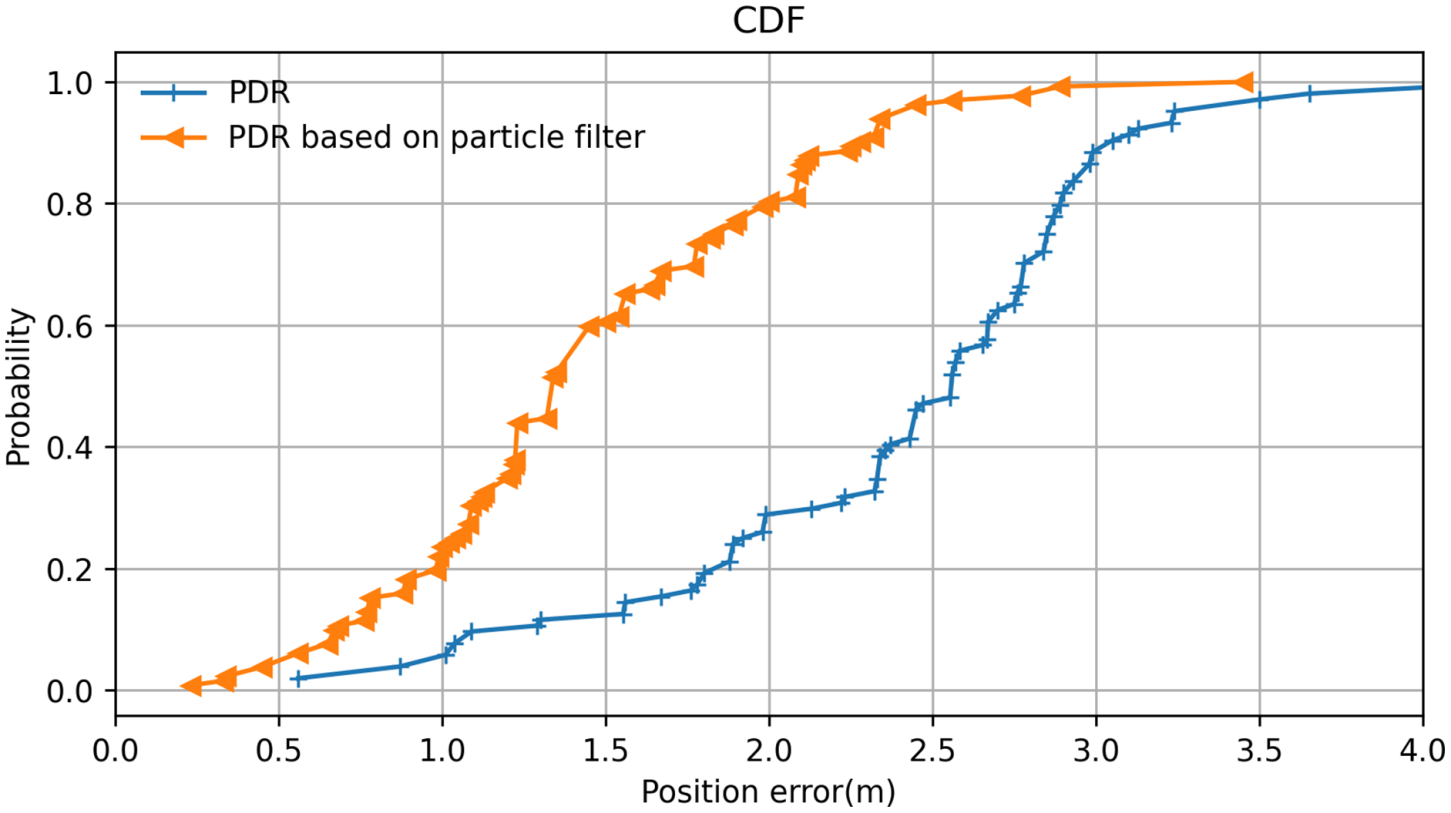
Positioning error analysis of the particle filter algorithm.

The resulting cumulative probability distribution of the positioning error is displayed in Fig. 14. It can be observed that PDR positioning with the particle filter is significantly better than PDR positioning without it. The positioning error range was greatly reduced by the inclusion of the particle filter algorithm, which improved the system’s positioning performance. At the same time, it was found that the probability of the error range was more than 80% within 2 m, which helps the PT camera to locate the lecturer when they move out of the camera’s view and greatly improves search efficiency. Moreover, because the camera itself has a certain angle of view, we concluded that the system effectively solves some failures associated with locating the lecturer caused by errors in the positioning system.

### Comparative analysis of related systems

The proposed system was then compared with existing related systems (Table 11). Multi-camera systems (Taj & Cavallaro, 2011) can be easily deployed for indoor and outdoor localization. However, for such a system to be effective, multiple cameras must collaborate, and the computing power of edge devices is high. Panoramic camera systems (Sun et al., 2020) are simple to deploy, but the associated image distortion needs to be corrected. In addition, although WIFI-PDR Integrated Indoor Positioning’s (Li et al., 2016) is highly accurate, it must collect WIFI offline information in advance. Camera-IR thermal sensors (Tan, Kuo & Liu, 2019) can satisfy the real-time demands with low cost, but it’s not easy to deploy. For instance, IR thermal sensors need to be fixed to the ceiling. When compared with Tan’s system, our proposed system has moderately better experimental results, and our system is portable and easier to deploy. The approach based on Siamese Fully Convolutional Classification and Regression (Guo et al., 2020) performs well in the public target tracking set. Nevertheless, it has significant equipment requirements for high performance, and the effect is not satisfactory in actual lecture-recording scenarios.

**Table 11:** Comparative analysis of existing lecturer tracking approaches.

Technology	Advantages	Disadvantages
Multi camera (Taj & Cavallaro, 2011)	Easy deployed on the indoor and outdoor localizations	Multi-camera fusion requires post-processing
Panoramic camera (Sun et al., 2020)	Convenient construction and low cost	Image distortion needs to be corrected
WIFI-PDR Integrated Indoor Positioning (Li et al., 2016)	Low cost, high positioning accuracy	Need to collect offline WiFi information and non-real time
Camera, IR thermal sensors (Tan, Kuo & Liu, 2019)	Low cost, real-time with good performance	Difficult to deploy, thermal sensors are sensitive to temperature
Siamese Fully Convolutional Classification and Regression (Guo et al., 2020)	Fast and accurate	High requirements for equipment performance
PT Camera and smartphone (the proposed system)	Portable, low-cost, strong robustness and easy to deploy	Only single-face detection may lead to temporary tracking failure

In conclusion, our proposed system is a better option for lecture recording than existing systems. Tracking accuracy has been greatly improved due to the introduction of PDR positioning technology with particle filtering. However, our system still has certain shortcomings. For example, it can only perform single-face detection; hence, when there are multiple faces in the picture, it cannot identify the faces. This topic warrants further study in future work.

## Conclusion and future work

We have proposed an intelligent lecturer tracking system that can be used for automated online video recording. The system integrates PDR information from a smartphone and visual information to detect the lecturer with high tracking accuracy. The system is suitable for real-time tracking, as the software can be deployed on a personal laptop.

The experimental results show that the PT camera can track the lecturer through face detection and recapture the lecturer when they unexpectedly move out of the camera’s field of view. Compared to existing systems, our system can track the target and use easily obtainable smartphone PDR information to retrieve a lost target. It is an economical and portable solution for lecturer tracking.

The PT camera rotation speeds are calculated from the lecturer’s current and predicted locations; however, this functionality is not yet perfect. In our future work, we plan to improve PT camera control to achieve better video quality. Another exciting direction for further research is the development of all the system’s modules for use on embedded devices, such as Raspberry Pi or FPGA, for a wide range of applications.

## Supplemental Information

10.7717/peerj-cs.971/supp-1 Supplemental Information 1 The system run video.

10.7717/peerj-cs.971/supp-2 Supplemental Information 2 System code and android app code.

10.7717/peerj-cs.971/supp-3 Supplemental Information 3 Particle filter raw data.

## Additional Information and Declarations

### Data Availability

The following information was supplied regarding data availability: The code is available in the Supplemental Files.
