# Supplementary figures and images for: Intelligent lecture recording system based on coordination of face-detection and pedestrian dead reckoning
Source: PeerJ Comput Sci. 2022 May 17;8:e971. doi: 10.7717/peerj-cs.971 (PMC9137921; doi:10.7717/peerj-cs.971)

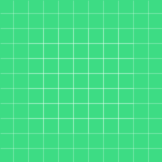

Supplement: Supplemental Information 2 [file peerj-cs-08-971-s002.zip › file2/app/build/generated/res/pngs/debug/drawable-hdpi/ic_launcher_background.png]

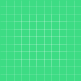

Supplement: Supplemental Information 2 [file peerj-cs-08-971-s002.zip › file2/app/build/generated/res/pngs/debug/drawable-ldpi/ic_launcher_background.png]

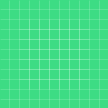

Supplement: Supplemental Information 2 [file peerj-cs-08-971-s002.zip › file2/app/build/generated/res/pngs/debug/drawable-mdpi/ic_launcher_background.png]

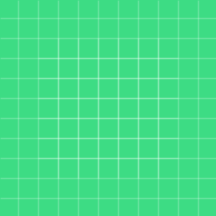

Supplement: Supplemental Information 2 [file peerj-cs-08-971-s002.zip › file2/app/build/generated/res/pngs/debug/drawable-xhdpi/ic_launcher_background.png]

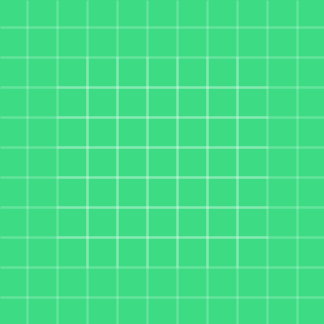

Supplement: Supplemental Information 2 [file peerj-cs-08-971-s002.zip › file2/app/build/generated/res/pngs/debug/drawable-xxhdpi/ic_launcher_background.png]

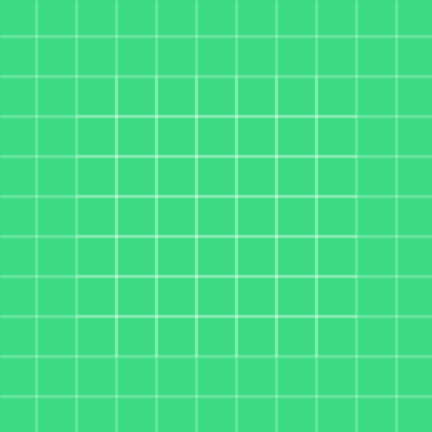

Supplement: Supplemental Information 2 [file peerj-cs-08-971-s002.zip › file2/app/build/generated/res/pngs/debug/drawable-xxxhdpi/ic_launcher_background.png]
